# Organizational Arrangements in Evidence2Success Communities: Enabling Sustainable Community Transformation for Youth Well-Being

**DOI:** 10.3390/soc16060169

**Published:** 2026-05-22

**Authors:** Jochebed G. Gayles, Sarah Meyer Chilenski, Mary Lisa Penilla, Sylvia Lin, Megan Galinsky, Francisco Villarruel, Patria Johnson, Charles Henderson, Jeremiah Newell

**Affiliations:** 1 Edna Bennett Pierce Prevention Research Center, College of Health and Human Development, The Pennsylvania State University, University Park, PA 16802, USA; 2 Human Development and Family Studies, College of Social Science, Michigan State University, East Lansing, MI 48824, USA; 3 Women’s Foundation for a Greater Memphis, Memphis, TN 38103, USA; 4 University Medical Billing-Operations, University of Utah Hospital and Clinics, Salt Lake City, UT 84111, USA; 5 Mobile Area Education Foundation, Mobile, AL 36608, USA

**Keywords:** asset-based community development, community coalitions, evidence-based prevention, youth well-being, community engagement, social capital, implementation science, community capacity building, data-driven decision-making

## Abstract

Building healthy communities requires organizational arrangements that center on resident and community assets while using data to guide decisions. This study examines how the Evidence2Success framework was implemented in three communities, Kearns, UT, Mobile, AL, and Memphis, TN, to understand how citizen-led asset mapping, coalition processes, and funding strategies shape youth well-being efforts. Using an interpretive case-study design, we analyzed process-evaluation interviews, implementation milestones and benchmarks, strengths-and-concerns reports, and community case materials to trace how coalitions mobilized assets, reoriented institutional resources, and adapted evidence-based programs. The results show that broad, cross-sector Community Boards completed most implementation tasks, increased participation by people of color, and developed more inclusive decision-making structures that addressed historical inequities. Coalitions also strengthened data-use capacities, employing youth survey results and local qualitative input to select priorities, braid funding, and make culturally responsive adaptations while maintaining program fidelity. Overall, the findings suggest that when evidence-based planning frameworks are embedded within asset-based, resident-governed structures, communities can build sustainable organizational arrangements that support youth well-being and advance more equitable local systems.

## 1. Introduction

Communities increasingly turn to data-driven planning to confront intertwined health, educational, and economic challenges, yet the success of such efforts still depends on mobilizing resident assets and shared purpose [1,2]. Evidence2Success supplies a rigorous, indicator-guided framework for selecting and funding evidence-based interventions [3–5]. However, little is known about how its on-the-ground implementation aligns with the long-standing principles of Asset-Based Community Development (ABCD). ABCD theory contends that sustainable change emerges when citizens map local strengths, weave social capital, and redirect institutional resources toward community-defined priorities [2,6–8]. Examining whether and how Evidence2Success operationalizes these principles can clarify the conditions under which evidence-oriented models remain authentically community-led [1,3,5,9]. Guided by an ABCD lens, we ask four questions: (1) How do citizen-driven asset-mapping activities within Evidence2Success shape local prevention priorities? (2) In what ways do coalition processes build horizontal and vertical social capital and foster inclusive governance? (3) How are institutional and economic resources reallocated to sustain resident-identified initiatives? Moreover, (4) How does resident knowledge inform program adaptation while preserving fidelity to evidence? Through case studies of three communities, we illuminate points of convergence and tension between Evidence2Success processes and ABCD principles, generating insights for scholars and practitioners seeking to marry empirical rigor with grassroots capacity. By illuminating where Evidence2Success processes converge with or diverge from ABCD principles, we clarify how evidence-oriented models can remain authentically community-led and inform both practice and theory.

Evidence2Success is best understood not as a single funded program, but as a structured community change framework that helps local coalitions use youth data, resident knowledge, and strategic financing to select and sustain evidence-based responses to locally defined priorities. In the communities examined here, the Annie E. Casey Foundation supported the broader initiative, framework development, and technical assistance infrastructure. At the same time, local implementation depended on resident-agency Community Boards, local partner organizations, and braided or reallocated funding streams assembled within each site. The Edna Bennett Pierce Prevention Research Center at Penn State played roles in framework development, process evaluation, action-guide development, and technical support. However, decisions about priorities, local adaptations, and program selection remained with community partners. This distinction is important for interpreting the cases that follow: the initiative provided structure, tools, and support, but community actors determined how those resources were translated into local prevention strategy and governance practice.

### 1.1. Conceptual Foundations

#### 1.1.1. Asset-Based Community Development (ABCD) Framework

Asset-Based Community Development emerged from Kretzmann and McKnight’s seminal work Building Communities from the Inside Out (1993), which critiqued deficit-oriented planning for reinforcing dependency on external aid [6]. ABCD reframes community change by asserting that every neighborhood already holds a reservoir of human, social, and material assets that can be mobilized from the “inside-out.” Early fieldwork showed that cataloging individual skills, local associations, and institutional resources not only counters deficit narratives but also seeds conditions for collective efficacy [2,7,8,10]. Central to the model is citizen leadership: residents are positioned as primary agents of change, while professionals play a supporting role. Systematic asset-mapping processes surface these capacities and, in turn, catalyze horizontal social capital among neighbors and vertical ties between residents and institutions. A growing body of evidence links these practices to heightened collective efficacy, civic participation, and program sustainability, demonstrating that communities thrive when development proceeds “inside-out” rather than “outside-in” [2,11]. Subsequent scholarship has elaborated four recurring asset domains: individual, associational, institutional, and physical that interact to generate social capital and drive locally defined action [8,11,12].

Beyond its methodological prescriptions, ABCD carries an implicit theory of social change. By foregrounding what communities have rather than what they lack, ABCD fosters a narrative of abundance that alters power relations between citizens and external actors [2,7,8]. This abundance narrative legitimizes resident knowledge, broadens the repertoire of locally acceptable solutions, and invites institutions to reorient their resources toward community-defined priorities. The process is iterative: as residents connect assets and act collectively, they generate new resources, skills, relationships, and material gains that feed back into the asset pool. Scholars have described this dynamic as a “spiral of assets,” in which early wins strengthen social cohesion, attract additional partners, and lay the groundwork for larger-scale transformation [2,10,13]. Accordingly, ABCD is not merely a toolset but a normative stance on empowerment and governance, positioning community strengths as the engine of sustainable development [6–8,10].

Empirical studies confirm that communities employing ABCD practices report higher civic participation, tighter resident networks, and greater program sustainability than those reliant on needs-based planning alone [2,14]. At the same time, ABCD research cautions that genuine resident leadership requires deliberate power-sharing with external agencies and continuous reflection on whose assets are recognized [15,16]. When these conditions are met, the framework produces a reinforcing cycle: asset-mapping builds trust, trust fuels joint action, and successful joint action enlarges the local stock of assets [2,13,17].

#### 1.1.2. Evidence2Success Framework

The Evidence2Success framework (the Framework) is a community-centric approach that integrates data-driven decision-making and preventive programming to support youth and families effectively [3,4,18]. Through the Evidence2Success initiative, the Annie E. Casey Foundation supports communities in identifying and monitoring local needs while employing evidence-based solutions tailored to those needs, thereby fostering a culture of continuous improvement and accountability [4,5,18]. The framework promotes the adoption of a results-based approach, where community stakeholders leverage empirical data to guide their strategies and evaluate their impacts, ultimately enhancing community resilience and capacity [3,18].

A critical aspect of Evidence2Success is its emphasis on building social capital and fostering resident ownership within the community ^1^. Research has shown that efforts to strengthen collective capability, such as improving community confidence in preventive measures and enhancing knowledge about available resources, are significantly correlated with positive community outcomes [3,4,9]. Conceptually, by engaging residents in co-creating programs and assigning them leadership roles, the Framework fosters a sense of ownership and empowerment, ultimately leading to sustained changes in the community [3–5]. Additionally, the Framework aligns well with the concept of collective efficacy, as it encourages citizens to participate actively in community change efforts, thereby fostering an environment conducive to the well-being of youth and their families [9,19]. In summary, Evidence2Success embodies a holistic, collaborative, and evidence-based approach to community development, aimed at fostering healthy environments conducive to positive youth development and community empowerment [3–5,9,19].

Grounded in implementation science and results-based accountability, the Framework unfolds in a five-phase cycle that couples rigorous data use with resident governance. Communities begin by administering the Youth Experience Survey (or a similar youth survey) and other indicator sets to identify priority outcomes [4,20]. A resident–agency Community Board then selects a portfolio of evidence-based programs matched to those outcomes, develops a financing strategy that braids public and private funds, and monitors implementation fidelity and results [4,5,20–22]. Technical assistance supports each stage, while decision-making authority, prioritization, action planning, and goal-setting remain local [3]. Continuous evaluation, including baseline assessments, quarterly feedback memos, and wave interviews, supplies real-time data that guide adaptation while safeguarding program fidelity. In short, Evidence2Success seeks to harmonize empirical rigor, equitable governance, and sustainable financing [3–5,9,19].

By grounding program choice in locally generated evidence, the Framework fosters “a culture of continuous improvement and accountability” in which empirical data guide both strategy and outcome monitoring [4,5,9]. Engaging residents as co-designers and decision-makers “strengthens collective capability” and is positively associated with community-level outcomes [3–5]. This participatory structure aligns with concepts of collective efficacy by inviting citizens to “participate actively in community change efforts,” thereby enhancing both empowerment and sustainability [9,19]. Technical assistance remains available throughout the five-year cycle to build capacity in data use, program selection, engagement tactics, and sustainability planning, ensuring that each community can adapt the model to its unique context while maintaining fidelity to evidence-based practice [4,21,22].

#### 1.1.3. Alignment Between Evidence2Success and ABCD

Although developed independently, Evidence2Success employs several mechanisms that echo and, in some respects, extend ABCD principles. While Evidence2Success was not explicitly designed as an ABCD model, many of its structures invite an asset-based reading. Both approaches elevate resident voice, rely on systematic discovery of local assets, and aim to redirect institutional resources toward community-defined goals. Evidence2Success adds explicit standards for evidence and a pooled-financing tool that helps communities scale interventions without relinquishing ownership [4,5,9]. Table 1 maps the ABCD principles summarized above onto the core components of Evidence2Success, illustrating points of convergence and extension.

Taken together, these alignments suggest that Evidence2Success operationalizes ABCD’s inside-out logic through structured data, governance, and financing routines, while extending the model by binding community assets to formal evidence standards and continuous-improvement cycles. The empirical sections that follow test how fully this convergence materializes in practice.

### 1.2. Current Study

This investigation examines how three Evidence2Success communities combine citizen-led asset discovery with an evidence-based strategic planning model. Four focal questions guide the study:

- How do citizen-driven asset-mapping activities within Evidence2Success shape local prevention priorities?
- In what ways do coalition processes build horizontal and vertical social capital and foster inclusive governance?
- How are institutional and economic resources re-allocated to sustain resident-identified initiatives?
- How does resident knowledge inform program adaptation while preserving fidelity to evidence?

Using an interpretive case study methodology [23,24], we explore how communities reconstruct and reimagine traditional power relationships through their implementation of the Framework ^2^. The three communities selected for this study represent diverse geographic, demographic, and socioeconomic contexts, ranging from urban centers to rural municipalities. These communities have implemented the Evidence2Success framework for at least two years, providing sufficient depth to examine both immediate implementation processes and longer-term sustainability factors. Through a systematic analysis of each community’s implementation journey, we investigate how local stakeholders integrated evidence-based practices to actualize core ABCD principles. The methodological approach combines qualitative data collection methods, including semi-structured interviews with key stakeholders, direct observation of community meetings and implementation activities, and analysis of program documentation and progress reports. Each community case study explores the unique contextual factors that influence the implementation of the framework, including local governance structures, existing community assets, and historical patterns of community engagement. This multi-layered analysis enables the identification of both common patterns and distinctive adaptations across sites. Each case study examines how social networks, cultural capital, and institutional arrangements influence the community’s capacity to implement evidence-based practices while maintaining local autonomy. The analysis particularly focuses on how marginalized voices are amplified or constrained within these implementation processes.

These qualitative materials are coded deductively for the five ABCD–Evidence2Success alignment constructs (citizen leadership, asset mapping,^3^ social-capital weaving, resource re-orientation, and continuous learning) and inductively for emergent themes that illuminate context-specific variation. Cross-case comparison then traces how each construct operates across divergent settings, highlighting patterns that answer the four questions above. By restricting data collection and analytic procedures to those explicitly recorded in the project documentation, the current study provides a grounded account of how communities leverage their own assets while meeting the evidence standards embedded in Evidence2Success [4,5].

## 2. Materials and Methods

### 2.1. Sample

The Evidence2Success framework was developed to establish or sustain community conditions that promote youth well-being and reduce racial inequities and health disparities, using a science-based, public health approach to prevention [4,5,9,21]. The framework was first implemented in Providence, Rhode Island, then expanded to include Selma and Dallas County, Alabama; Mobile, Alabama; Kearns Township and Salt Lake County, Utah; Memphis, Tennessee; and Miami, Florida [4,5,9,22]. Three of the six communities are examined using a case-study approach. These include Kearns, Memphis, and Mobile.

### 2.2. Study Procedures

The Evidence2Success framework (the Framework) harnesses learnings from prevention science and collective impact to bring public systems and resident leaders together to improve child well-being [1,3,4,9,18,21]. Communities identify priorities by using data from youth about risk and protective factors and by elevating local lived experience and wisdom to inform the selection of tested, effective programs to address priority outcomes [4,20]. The Framework, adapted from the Communities that Care (CTC) system, is founded on core, data-centric strategies: a broad, cross-sector governance structure that includes public system representatives, local leaders, and residents of focus neighborhoods; data use to make decisions and develop strategies; comprehensive financing strategies; implementation of tested, effective programs; and performance measures [4,5,9,14,18,22]. Evidence2Success uses the same five phases of CTC described in prior prevention-science work: getting started, getting organized, developing a community profile, creating a plan, and implementing and evaluating that plan [14–17].

In Phase 1, Get Started, the community and foundation agree to jointly implement Evidence2Success and sign a memorandum of understanding to demonstrate acceptance of the terms [4,14,21]. Phase 2, Get Organized, includes forming a diverse, effective, and cohesive coalition, led by a local coordinator or facilitator [9,21]. Phase 3, Developing a Community Profile, involves using youth survey data to assess community-wide levels of youth well-being, problems, risk, and protection. Then, the community coalition uses these data to prioritize needs; communities use a survey to gather data directly from youth, measuring risk and protective factors as well as adolescent behavioral health [9,21]. The coalition also examines gaps in current programming addressing the prioritized outcomes and risk and protective factors [4,9]. In Phase 4, Create a Plan, the coalition establishes community outcome goals related to priorities established in the previous phase and then selects tested, effective interventions, including programs, policies, and practices, to address their priorities [4,5]. In Phase 5, Implement and Evaluate, the coalition plans for and oversees implementation of the selected interventions, ensuring that they are implemented with fidelity and reach [4,5,9,22]. This process continues cyclically, with communities revisiting data, priorities, and program portfolios over time [4,5,9,22].

The logic model, presented in Figure 1, theorizes how the Framework proceeds to make systems-level changes for youth and family well-being. Communities use inputs to carry out activities that produce short-term outputs, intermediate outputs, and, ultimately, outcomes related to youth and family well-being. Attention to racial and ethnic equity and inclusion (REEI), prioritizing the reduction in disparities, grounds the entire process and outcomes [18,22]. Framework implementation drives changes in how the communities and focused neighborhoods make decisions about, plan for, implement, and evaluate youth and family programs that can positively impact outcomes for kids and families and create “a new way of doing business” in communities. The logic model guided data collection for the process evaluation. When the framework changed, the evaluation process was updated [4,5,9,22].

### 2.3. Measures

#### 2.3.1. Case Study

A case study approach is a research method for in-depth investigation of complex issues and their associated dimensions in real-life contexts [24,25]. Three of the six communities were closely examined, and case studies were written to describe how they collaborated to collect, review, and interpret data. First, a general narrative about the community and its overall makeup was written, noting demographics, key groups, and geographic specifics. Next, the community board describes who they are, who was at the table doing the work, and how diverse or representative the board was. A brief history is provided of how decisions were made before Evidence2Success entered the picture: Who traditionally held decision-making power? What was the broader context the community was operating in at the time? Additionally, how data was used (or not) in past decision-making, and whether the broader community was engaged in that process, are described. After setting the stage, the process is described.

This process section explains what data the community collected and reviewed, which may include youth outcomes, board functioning, or other local change indicators [4,19,22]. Questions asked in this section include: What did the process actually look like? The structure and frequency of meetings, who participated, who facilitated, and how feedback was collected were documented. The kinds of questions asked by stakeholders were reflected upon, input was gathered from various contributors, and attention was given to identifying who was actively listened to [9,19,22]. The unfolding of the co-engagement process was detailed, with emphasis on its intentionality, the modeling demonstrated, and the level of community ownership observed [5,22,26,27]. Consideration was given to whether reflective or reflexive practices had been embedded and, if so, in what forms. Any “aha” moments that emerged during data interpretation were noted. How board members engaged meaningfully with the data were described, and key moments and insights were illustrated through quotes drawn from interviews with communities and the transcripts [4,5,22,26,27].

The outcome section captures the essence of the case study data reviewed, highlighting the overall theme of community stakeholders using, reviewing, interpreting, and acting on data insights. Questions in this section include: What insights were identified during data interpretation? How were these insights translated into action? What things were observed in terms of change [18,21,25–27]?

#### 2.3.2. Interview

One-hour qualitative interviews were scheduled with eight community leaders from the six Evidence2Success communities as part of our community-facing Guiding Collective Change Series [4,5,9,19,20]. These interviews took place in June and July 2024. The purpose of the interviews was to understand how communities adapted the Evidence2Success framework to meet their unique needs. Notes and transcripts from these interviews were thoroughly examined and thematically coded to identify key patterns and perspectives around adapting the community change effort to local culture and context. Direct quotes were selected to highlight pivotal moments and illustrate the importance of adaptation, including the role of data in this process. Each case study was constructed to reflect not only the outcomes of Evidence2Success but also the reflective and reflexive processes that drove change in the community [4,5,9,19,20,22].

#### 2.3.3. Strengths and Concerns Reports

The Strengths and Concerns reports highlight communities’ successes and challenges in implementing the Framework, as well as recommendations for moving forward. The process evaluation team wrote reports for each community at varying intervals (from monthly to quarterly), based on Coach/TA notes, implementation progress interviews, workshop evaluations, meeting checkup data, and information directly from the communities [5,22,26,27]. Each report was shared with the technical assistance providers working with the reviewed community to inform coaching calls and planning. Additionally, the reports captured progress over time while documenting the Evidence2Success process [5,22,26,27].

Case study authors reviewed the Strengths and Concerns reports to understand how communities used data and to add details about participants’ experiences, including quotes, on how they used the Evidence2Success framework to further youth well-being in their communities.

#### 2.3.4. Implementation Progress Interviews

Communities participated in three waves of interviews, starting at the end of Phase 2 and occurring approximately annually through year 3 [22]. Interviews with community board members and key leaders asked more than 100 Likert-style and open-ended questions, covering key areas related to the adoption and implementation of the revised Evidence2Success framework [22,26]. Domains included participation in the initiative, anticipated and past challenges, leadership, community engagement, shared ownership, collaboration, the use of tested and effective prevention programs, and the integration of a science-based decision-making process [22,26]. Case study authors examine quotes from open-ended questions and incorporate interview details to develop the case study [21,25].

At each wave of the Implementation Progress Interview, participants were asked to select which sector they identified with. The comprehensive list was then recoded into the following categories: community, education, health, industry, other government, law, social services, and other. Additionally, the survey ends with a demographics section. One of the questions asks about race and ethnicity. The interview includes an inclusive list of races and ethnicities, which were recoded into White and People of Color [21,25].

#### 2.3.5. Milestones and Benchmarks Ratings

Every six months, communities rate and report progress towards completion to the process evaluation team. The tool outlines the key steps and sub-steps of the Evidence2Success process in a checklist format, allowing users to track progress toward completion and fidelity to the process [4,5,22,25]. It is designed to assist the coordinator, working with the community board, in tracking several streams of effort across five phases of work. In Evidence2Success, the Milestones and Benchmarks tool helped communities determine when to transition to the next phase. Sequential, actual implementation may vary due to local conditions, culture, or other contextual factors—percent complete of each phase by community and overall. For this study, the average completion of each phase was calculated by community and for the overall Evidence2Success mean [4,19,20,22,25].

## 3. Results

### 3.1. Community Context

#### 3.1.1. Kearns, Salt Lake County

Kearns, a township in Salt Lake County, Utah, was a diverse community of about 36,530 residents during the early Evidence2Success period. Originally developed as a World War II–era military housing area, Kearns evolved through periods of focused investment, most notably the construction of the Utah Olympic Oval for the 2002 Olympic Games, followed by longer stretches of disinvestment. In 2015, Kearns had an overall poverty rate of approximately 9%, one of the highest child poverty rates in Utah, and a high rate of intergenerational poverty. Its population tended to be young, with the county’s largest average household size and substantial ethnic diversity, including a significant Hispanic population alongside non-Hispanic White residents, as well as a mix of longtime families and newcomers [28,29].

Youth survey data and related indicator profiles highlighted concerns about school engagement, depressive symptoms, and neighborhood conditions. For example, youth in the Kearns focus area reported lower perceptions of neighborhood safety and higher levels of emotional distress than county averages, underscoring the need for supports that addressed both environmental and socioemotional risks. At the same time, local media and community narratives emphasized Kearns’s latent strengths: an emerging culture of cross-agency collaboration and a growing recognition among residents and leaders that “extraordinary efforts” by dozens of government agencies and community leaders could reverse distressing statistics for children and youth. These conditions made Kearns a compelling site for testing how Evidence2Success could align formal systems with resident-defined assets and priorities (Strengths and Concerns reports for Kearns) [5,9,20].

#### 3.1.2. Mobile, Alabama

Mobile, Alabama, is a racially and ethnically diverse, mid-sized city in southwest Alabama with roughly 201,000 residents at the start of Evidence2Success implementation. In 2013, family poverty in Mobile stood at nearly 20%, with Black, Hispanic, and Pacific Islander residents experiencing disproportionately high rates, and poverty was geographically concentrated in specific census tracts. Three neighborhoods were selected to launch Evidence2Success: Maysville, Lower Dauphin Island Parkway neighborhood (LDIPN) and Martin Luther King, Jr. neighborhood (MLKN). LDIPN consisted of three census tracts and was home to approximately 9500 residents, with a 2013 poverty rate of 26%; MLKN comprised five census tracts, roughly 8900 residents, and a 2013 poverty rate of 44% [30].

Although Mobile had a history of developing broad-based citywide collaborations, those efforts rarely reached down to the targeted neighborhood level or fully incorporated residents from high-poverty areas into agenda-setting and budgeting processes. Early YES and administrative data pointed to elevated levels of youth violence exposure, chronic absenteeism, and low perceptions of community cohesion in the focus neighborhoods. At the same time, asset mapping documented dense informal networks—churches, historically Black Greek organizations, neighborhood associations—that could be mobilized to support group mentoring and other relationship-based interventions. Evidence2Success entered this landscape as a structure to connect long-standing collaborative aspirations with neighborhood-level resident leadership and data-informed decision-making (Strengths and Concerns reports for Mobile) [4,5,9]. The role of Evidence2Success in aligning community assets and planning priorities in Mobile is detailed in the Action Guides [4,9].

#### 3.1.3. Memphis, Tennessee

Memphis (population 650,932) anchors a larger metropolitan region in Shelby County (population 938,069 in 2015). The Evidence2Success focus area, South City, encompassed approximately 5868 residents across more than 115 city blocks (about 880 acres, or 1.37 square miles) directly southeast of the city’s Central Business District. South City includes Foote Homes (roughly 414 households and 1038 individuals). Front Street, Beale Street, Walker Avenue, Crump Boulevard, a railroad right-of-way, Main Street, Kansas Street, and Georgia Avenue bounded it. Characterized by substandard and subsidized housing, high unemployment, limited educational institutions, constrained access to health care, and elevated violent crime, South City has been identified as the poorest area in Memphis and meets the federal Choice Neighborhoods Initiative definition of “severely distressed” [31,32].

Socioeconomic and demographic indicators underscore the depth of disadvantage. South City’s poverty rate is approximately 64.6%, with long-term residential vacancy near 12.4%, compared to 7.4% in Shelby County. The average annual violent crime rate in South City (2011–2013) was 21.8 per 1000 residents, versus 13.16 per 1000 in Memphis overall. The population is overwhelmingly African American (about 84%), compared to 63% in the city and 45.6% in the broader metropolitan statistical area; Whites comprise 12.5% of the South City population, compared to 30.4% and 48.4% in the city and MSA, respectively. 64% of residents are between ages 18 and 44, and only 3% are 65 or older, a markedly younger age profile than that of the city and region. Median household income in South City is roughly $11,350, with 65.3% of residents living below the poverty line, in stark contrast to Memphis ($36,912; 26.9% poverty) and the MSA ($47,497; 19.2% poverty). Unemployment in South City (27.2%) is nearly twice that of the city (14.4%) and MSA (11.5%) [33].

Despite these indicators, South City also contains significant assets. The neighborhood is anchored by iconic institutions such as the National Civil Rights Museum, Memphis Central Station, and the Memphis Heritage Trail. It has a rich African American history and culture that residents and leaders view as central to community identity. Recent housing and commercial developments, the availability of vacant land for redevelopment, and proximity to downtown and the medical district position South City for reinvestment if local stakeholders can harness those opportunities in ways that center existing residents. Within Evidence2Success, Memphis and the REEI work treated race, ethnicity, equity, and inclusion as cross-cutting priorities, using YES data, neighborhood indicators, and youth-led, image-based documentation used alongside survey and indicator data to frame South City not only in terms of risk, but also in terms of community strengths and structural inequities that needed to be addressed through organizational and systems change [19,22].

### 3.2. Study Fundings

Qualitative data consisted of semi-structured leadership interviews with community board site coordinators (Mobile, Kearns) and the site and finance coordinators (Memphis). Interviews were transcribed and coded thematically using an iterative, constant-comparison process that combined a priori codes derived from the four research questions with inductive codes capturing emergent concepts within and across sites. Coding and theme development focused on cross-case patterns in governance, financing, and local adaptation, while also preserving site-specific nuances; the tables that follow present illustrative quotations for key themes, rather than an exhaustive set of coded segments ^4^.

Across the three sites, the findings converged around four interrelated dynamics: asset discovery helped coalitions translate broad concern into concrete prevention priorities; participatory governance practices built both horizontal and vertical social capital; transparent financing processes enabled the reallocation of existing resources; and resident interpretation of data shaped culturally responsive adaptation without abandoning fidelity to evidence-based models. Rather than treating these as isolated steps, the cases show how they worked together in a reinforcing cycle through which resident voice became more consequential in local decision-making and, in turn, more clearly connected to youth well-being goals ^5^.

#### 3.2.1. RQ 1: How Do Citizen-Driven Asset-Mapping Activities Within Evidence2Success Shape Local Prevention Priorities?

Across Kearns, Mobile, and Memphis, asset-mapping served as the catalytic event that moved each coalition from broad concern to specific, prevention-focused goals [9,19]. In Kearns, coalition members supplemented youth survey findings with local knowledge about youth-serving organizations and informal supports, including school-based and community-based activities, to better understand existing strengths and resources (Strengths and Concerns reports for Kearns) [9,19]. Coalition members overlaid these findings on their youth survey data indicator profiles, which showed elevated depressive symptoms and low perceptions of neighborhood safety among adolescents, revealing a gap in evidence-based programs for early adolescents, revealing a gap in evidence-based programs for early adolescents, ages 11–13 (Resource assessment Workshop documentation; Strengths and Concerns reports for Kearns) [20]. Kearns’ Community Board (i.e., MyKearns Coalition) used these analyses to initially select an evidence-based parent program, Guiding Good Choices, and to later select a youth-focused skills-building program, Blues, to address their highest-priority risk and protective factors. Through the Evidence2Success strategic financing and fundmapping ^6^ process, the Kearns coalition and its county partners examined how existing juvenile justice and other youth-serving budgets were being spent in the township, identified misalignments with priorities surfaced by the youth survey data and residents, and agreed to realign portions of existing funds toward evidence-based youth and family programs in Kearns, facilitating quick implementation of selected programs without new appropriations [5,19].

In Mobile, asset-mapping was embedded in community-facing data sessions where residents annotated YES findings and other information with Post-it notes describing local strengths. The process surfaced a rich network of church-based mentors and neighborhood organizations, prompting the coalition to shift resources from primarily individually oriented counseling toward group mentoring and other relationship-building formats that could leverage these endogenous supports [9,27]. Residents described noticeable changes, including more communication, greater neighborhood involvement, and increased youth participation, emphasizing that adults wanted to support their kids. The Mobile coalition then used its asset map and YES risk/protection data jointly to prioritize mentoring and relationship-building interventions that aligned with these community-identified strengths over more clinic-based models (Mobile Community Assessment report, Agency Fundmapping data meeting; Strengths and Concerns reports for Mobile) [4,9,27].

Memphis drew on fund-mapping and Youth Experience Survey (YES) data, along with other local information about neighborhood conditions, to develop an asset map for South City informed by resident stories [9,19]. Storytelling was a way to get youth to express themselves about trauma, and this information was helpful to inform which risk and protective factors to prioritize in the South City Community Action Plan. These findings helped stakeholders move beyond a narrow focus on traditional classroom-based substance-use curricula toward programs that emphasized family relationships and youth mental health, including Strong African American Families (SAAF) and Cognitive Behavioral Intervention for Trauma in Schools (CBITS), which they selected to address priorities that had emerged for South City youth and families (Memphis Fundmapping Data meetings data summaries) [19]. In keeping with Evidence2Success guidance on youth surveys, Memphis stakeholders treated YES findings on community risk and youth wellbeing as starting points for conversation, then combined those data with local qualitative insights and coalition deliberation to refine which risk and protective factors to prioritize in South City’s action plan [9,19].

Illustrative quotations from each community highlight how pre-existing coalitions and local data collection jointly shaped these prevention priorities (Table 2).

#### 3.2.2. RQ 2: In What Ways Do Coalition Processes Build Horizontal and Vertical Social Capital and Foster Inclusive Governance?

All three sites reported growth in horizontal social capital ties among resident groups through participatory planning routines. In Kearns, the coalition emphasized full-board deliberation on priorities and overall direction, while using smaller groups to prepare agendas and carry out specific tasks like completing the action plan. Evaluation data and interviews indicate that resident participation in coalition meetings increased between Year 1 and Year 3, and respondents described the board as a key place where community decisions about youth well-being were made (Strengths and concerns reports for Kearns) [9,19].

Mobile’s coalition emphasized inclusive governance and began strengthening youth involvement in decision-making about priorities and programs, including renewed efforts to organize with youth rather than relying only on adult leaders [9,19]. Coalition leaders emphasized that engaging school district leadership and bringing residents to the same table with public systems were critical for building trust and sustaining collaboration across systems. Engagement data from the cross-site fidelity assessment show that ratings of racial inclusiveness and participation improved by more than 20% between Year 1 and Year 3 across Evidence2Success communities [9]. In Mobile, this broader trend was reflected in a community board that increasingly engaged public leaders and residents, including a high-profile kickoff and key leader orientation with the mayor and system heads, growing collaboration with Mobile County Public Schools using YES data, and more consistent board meetings by 2018 with roughly 15 regular participants (Strengths and Concerns reports for Mobile) [27].

Memphis focused more explicitly on vertical ties through its Community Board and relationships with Shelby County Schools to connect neighborhood priorities to system-level decisions, including the district’s interest in administering the YES survey more broadly and aligning selected programs such as CBITS with district initiatives (Strengths and Concerns reports for Memphis). Horizontal bridging was visible in Mobile, as resident and agency partners worked together through fund-mapping and strategic finance planning to redirect existing public resources, including school-district dollars, toward community-selected programs and school-based prevention efforts (Strengths and Concerns reports for Mobile) [19]. Vertical linking was also evident in Kearns, where the finance staff member “led a sustainability workgroup that regularly reviewed detailed budget reports,” which helped the board secure direct budget-line commitments from three county departments after presenting asset-map gaps and YES indicators to the mayor’s cabinet.

Table 3 provides illustrative quotations that show how these governance structures and practices built both cross-sector collaboration and space for a more equitable resident voice.

#### 3.2.3. RQ 3: How Are Institutional and Economic Resources Re-Allocated to Sustain Resident-Identified Initiatives?

Resource re-orientation unfolded in staged increments, often starting with small, low-risk reallocations before progressing to larger budget shifts. (Kearns) Kearns used the Evidence2Success strategic financing process and fund-mapping across multiple county agencies and the school district to identify how existing youth-service juvenile-justice dollars were being spent, then began shifting, towards initially selected evidence-based prevention programs for middle-school youth, in line with priorities derived from youth survey data and community input [5]. (Mobile) Mobile used fundmapping and city-level partnerships to leverage existing public and philanthropic resources for group mentoring, targeting additional focus neighborhoods identified through YES data and local asset-mapping as needing stronger l youth supports (Strengths and Concerns reports for Mobile) [19]. (Memphis) Drawing on the strategic financing process, Memphis developed a braided-funding model in South City that combined school district support for CBITS, Community Development Block Grant funds for Strong African American Families, and philanthropic investments from the Kresge Foundation, the Women’s Foundation for a Greater Memphis, and an anonymous donor, using fundmapping to redirect existing public and private resources toward community-selected evidence-based programs (Memphis Fundmapping data summary; See Table 4) [5].

Coalition members emphasized that transparent fundmapping and strategic financing work both depended on and helped build trust by making funding information more visible across partners. In Mobile, leaders described the fund-mapping and strategic-financing sessions as a way to put funding information from multiple partners on the table, so they could see where money was already being spent and discuss whether existing dollars could be aligned with what young people and residents said they needed, rather than always looking for a new grant. They also noted that without concrete tools and follow-through, long meetings and complex processes made it easy for partners to lose focus or for priorities to slip down crowded agendas, which is why finance maps and written action plans were important for sustaining attention to youth priorities (Strengths and Concerns reports for Mobile) [5,19]. Memphis stakeholders similarly described how documenting funding sources and responsibilities through the fundmapping and strategic finance process, including entering more than fifty programs into a shared fundmapping database and using one-on-one meetings to secure funding commitments, clarified which organizations were contributing which dollars and for what purposes. Memphis finance leaders noted that this shared database was “a potentially useful tool and a valuable resource for the community” and that fundmapping and strategic finance planning invited agencies to shift their funding mindset toward reallocating existing dollars to tested, effective programs rather than maintaining separate, siloed efforts (Strengths and Concerns reports for Memphis). Cross-site Milestones and Benchmarks data indicate that, by Year 3, all Evidence2Success communities had completed over 75% of key financing tasks such as identifying funding streams, aligning budgets with selected programs, and planning for sustainability, suggesting that finance structures had been institutionalized beyond initial grant cycles [22,25].

Table 5 illustrates how leaders across sites used strategic financing and fundmapping tools both to surface reallocation opportunities and to confront the limits of braided, short-term funding.

#### 3.2.4. RQ 4: How Does Resident Knowledge Inform Program Adaptation While Preserving Fidelity to Evidence?

All three coalitions adopted formal feedback loops, quarterly Strengths and Concerns memos, “fidelity huddles,” and periodic implementation-progress interviews, but resident insight drove the most substantive adaptations [19]. Across sites, “the collected data was interpreted by local leaders, including resident adults and youth, to understand what was important to the community,” and coalition-level collaboration “led to increased funding towards community-selected priorities and programs that reflected community culture and context” [19]. In Kearns, parents recommended adding bilingual facilitators for Guiding Good Choices to address language barriers and cultural concerns; fidelity monitoring showed no compromise to core components, and post-adaptation session attendance climbed by 23% [27].

Mobile’s mentoring program attended to cultural heritage after a lack of historical context and opportunities to connect with Black community leaders was flagged. An external evaluator reported that the revised curriculum “retained the program’s evidence-based structure and dosage while increasing relevance and engagement for participating youth,” noting stable fidelity scores alongside improved attendance [25,26]. Memphis adjusted the dosage of after-school CBITS sessions infused with art therapy from 90 to 60 min when youth cited “after-school bus-run conflicts,” maintaining fidelity thresholds by adding two extra sessions to the cycle and preserving the total number of contact hours [25].

Across sites, technical assistance providers and coalition leaders observed that adaptations were most successful when framed as responses to documented barriers, such as participation, transportation, or schedule constraints, rather than as individual preferences. Communities routinely drew on multiple forms of evidence, including youth survey results, meeting-feedback tools, and locally generated indicators, to interpret needs and justify changes with program purveyors and technical assistance providers. These patterns echo broader Evidence2Success guidance that encourages coalitions to use fidelity and planning tools iteratively, preserving the core components of tested programs while adapting peripheral elements, such as format, scheduling, or engagement strategies, in order to fit local culture, history, and context.

Table 6 presents exemplar quotations that show how implementer and resident feedback shaped local adaptations, as well as how leaders themselves described ongoing gaps in resident engagement.

Together, these findings show how asset-based, resident-driven processes within Evidence2Success can translate local knowledge into prevention priorities, build cross-sector and resident social capital, realign public and philanthropic spending, and support culturally responsive adaptations without eroding evidence standards.

## 4. Discussion

This research contributes to sociological understanding of how communities navigate institutional and community power structures while pursuing social transformation by tracing how asset-based, resident-driven processes operate within an evidence-based framework. By examining the intersection of evidence-based frameworks with asset-based approaches, the study illuminates how communities can leverage institutional resources, data tools, governance structures, and public and philanthropic funds, while maintaining their agency in social change processes. The findings advance both theoretical understanding of community-based social change and practical knowledge for implementing evidence-based frameworks in ways that enhance rather than diminish community power.

### 4.1. Synthesis of Findings Vis-À-Vis ABCD Theory

Across the three Evidence2Success communities, our findings show that the framework can operationalize core Asset-Based Community Development (ABCD) principles when resident leadership, asset-mapping, and shared governance are intentionally foregrounded [1,2,13,34–37]. Asset-mapping activities in Kearns, Mobile, and Memphis functioned not only as technical exercises but as relational practices that surfaced informal supports, re-situated communities as capable, and built the trust needed for coalitions to take risks with resource reallocation. In this sense, Evidence2Success helped communities move from a deficit-oriented logic of “fixing problems” toward an abundance narrative more consistent with ABCD’s emphasis on local strengths and citizen agency [2,6–8,10,11,35,36].

At the same time, the case studies highlight tensions between the evidence standards embedded in the framework and ABCD’s insistence on bottom-up control, echoing debates about how to generate robust evidence for asset-based work without undermining community-led governance [2,34,36–41]. In several instances, resident-identified priorities (for example, group mentoring formats or placemaking activities) had to be translated into evidence-based program options that met funder expectations, creating pressure to fit local aspirations into existing intervention catalogs. These dynamics echo critiques that, without real community control over agenda-setting and resource decisions, asset-based approaches can be co-opted to further institutional agendas if communities lack real decision-making power over financing and program choices [1,15,37,39]. Our cases suggest that these tensions are most productively navigated when coalitions use asset and finance maps as boundary objects that allow residents and system leaders to negotiate trade-offs transparently. This finding is consistent with boundary-object theory and empirical work showing that shared artifacts, such as maps, can connect different “social worlds” while preserving local meanings [42,43].

### 4.2. Implications for Community Prevention Practice and Policy

For practitioners, the cases underscore that frameworks like Evidence2Success are most powerful when implemented as vehicles for community governance, aligning with evidence that collaborative governance and communities of practice are most effective when they center local partners in decision-making [1–3,9,22,44–47]. In Evidence2Success communities, coalition processes that centered resident voice and built social capital over time were associated with stronger collaboration and problem solving, consistent with emerging reports that Evidence2Success coalitions make progress when they attend to community context, diverse representation, and trust-building [3,9,19,22,45]. Coalition processes that institutionalized resident roles in agenda-setting, budget decisions, and adaptation “fidelity huddles” were better able to realign existing public funds and sustain programs beyond initial grant cycles, echoing both the Evidence2Success financing lessons and broader implementation guidance on balancing fidelity and adaptation. Practitioners should therefore invest early in structures that embed resident representation (including youth and caregivers) in formal decision nodes, such as rotating co-chair positions, community-held voting seats, and shared finance reviews, an approach consistent with guidance on creating resident-led governance structures and on collaborative governance models for building trust with marginalized communities [1,2,5,9,44,47,48].

For policymakers and funders, the findings point to the importance of designing evidence-use requirements that recognize locally generated knowledge as a legitimate form of evidence, in line with recent guidance on engaging lived experience in policymaking and recognizing practice-based and experiential knowledge as part of the evidence base [22,38,40,49–51]. When resident observations, qualitative data, and lived experience were explicitly integrated into decision criteria, communities were more willing to repurpose existing dollars and to adapt interventions to cultural and logistical realities without compromising core components; this echoes implementation guidance on balancing fidelity and adaptation, and with social work reviews documenting common, planned adaptations to improve contextual fit [3,9,19,52–54]. Policy guidance that encourages adaptive implementation, provides flexible funding for relational work (e.g., walks, listening sessions, youth photovoice), and protects resident seats on governing bodies can help ensure that evidence-based prevention strengthens, rather than supplants, community power [9,53,55–59]. To document and learn from adaptations while safeguarding fidelity to core components, teams can apply frameworks for characterizing fidelity and adaptations and draw on scoping reviews that show how social work interventions are routinely adapted to better fit local contexts [3,13,24,25,49,60].

### 4.3. Limitations and Future Research

Several limitations qualify these conclusions. First, the analysis relies primarily on qualitative case study materials and internal process data from three communities that volunteered or were selected for early adoption of the framework, limiting generalizability to other settings or later implementations. Similar challenges are noted in evaluations of community-level initiatives and asset-based approaches in public health [38,60]. Second, many of the quantitative indicators (e.g., attendance trends, budget reallocation percentages) were collected for internal learning rather than for research and may be incomplete or measured inconsistently across sites. These are issues that mirror the difficulty of generating economic and outcome evidence for asset-based approaches [44,49,61,62]. Third, our analytical focus on formal coalition structures may understate the contributions of informal actors and networks operating outside the Evidence2Success governance architecture; this limitation has been commonly highlighted in other community development studies [53,63–65].

Future research could extend this work by linking community-level implementation constructs (such as asset-mapping intensity, resident voting power, and financial transparency) to longitudinal outcomes in youth well-being, equity, and system change, building on frameworks that differentiate the “degree of asset-basedness” and offer comparable indicators across cases [3,13,24,25,49,60]. Mixed-methods comparative studies across a larger number of communities would allow testing hypotheses about which organizational arrangements are most strongly associated with sustained shifts in resource allocation and decision-making authority. Inquiry with a larger number of communities aligns with calls for more rigorous yet context-sensitive evaluations of community-level initiatives [44,49,61,62]. Additionally, participatory research led by residents and youth could deepen understanding of how frameworks like Evidence2Success are experienced “from below” and what communities themselves consider markers of success. This work would build on youth photovoice evidence that advances critical awareness and policy engagement among populations in diverse and resource-limited settings [53,63–65].

Importantly, the value of these organizational arrangements is not procedural alone. When residents, youth, and local partners exercised meaningful influence over priority-setting, implementation supports, and funding decisions, the resulting prevention strategies were better aligned with the realities shaping young people’s safety, belonging, and developmental opportunities. In that sense, governance quality functioned as part of the youth well-being intervention itself, because it shaped which problems were defined, which assets were mobilized, and which solutions were sustained over time.

## 5. Conclusions

These case studies demonstrate that evidence-oriented frameworks and asset-based approaches need not be in tension when residents hold meaningful authority over how data are interpreted, how assets are connected, and how resources are deployed. In Kearns, Mobile, and Memphis, Evidence2Success functioned most effectively when it operated not simply as a technical planning model, but as a resident-engaged governance structure that helped communities align local knowledge, institutional relationships, and financing decisions around youth well-being. The cases also show that youth well-being is not only an outcome at the end of implementation; it is shaped throughout the process by who participates, whose knowledge counts, and whether coalitions can adapt evidence-based strategies to community realities without losing fidelity. For scholars, practitioners, funders, and policymakers, the findings suggest that sustainable community transformation is most likely when rigorous data use is paired with organizational arrangements that keep resident leadership, equity, and community assets at the center of prevention work.

## Figures and Tables

**Figure 1.**
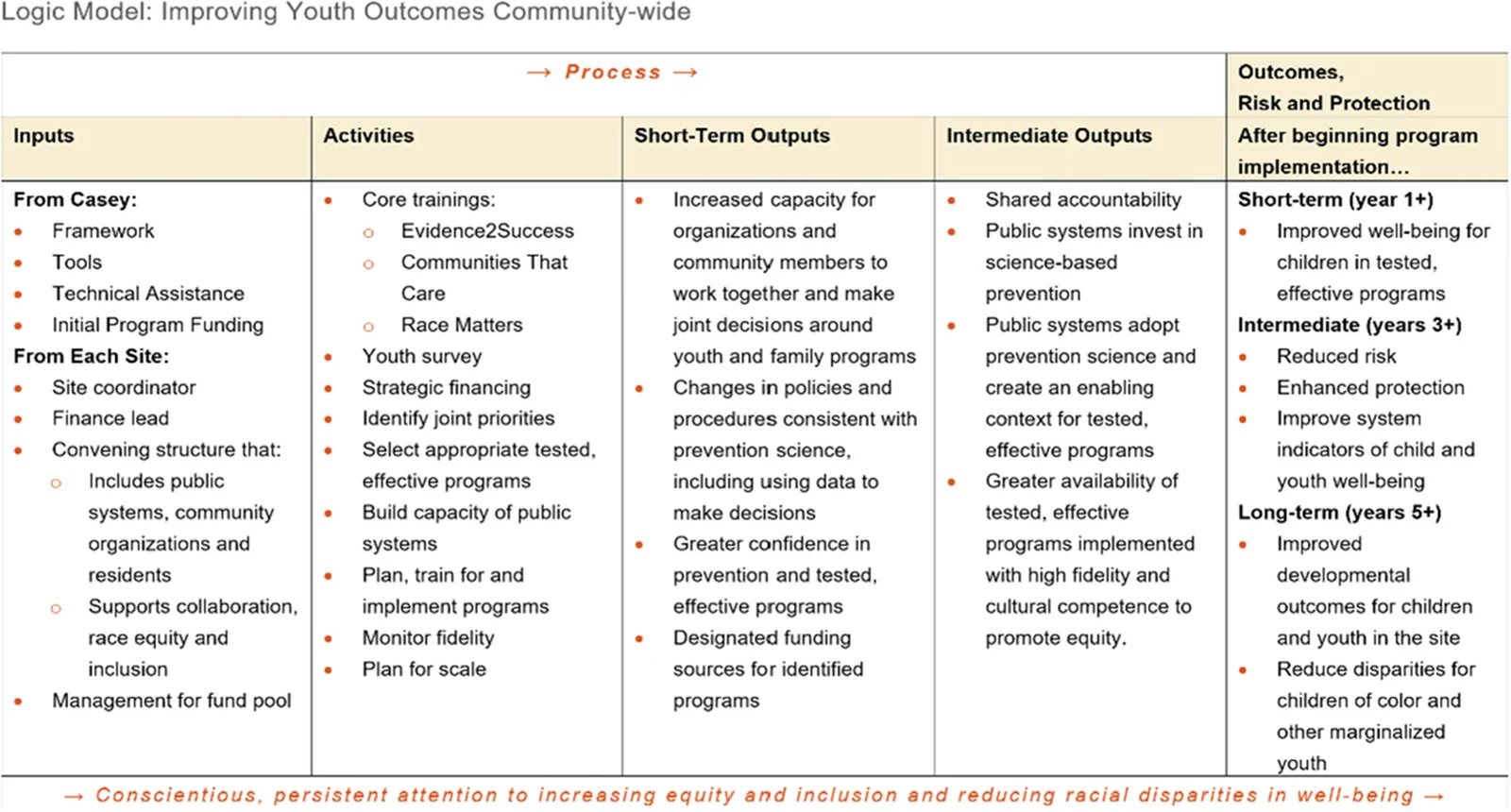
Evidence2Success logic model showing the flow from inputs (Casey Foundation framework, tools, technical assistance, and site-level resources including coordinator and finance lead) through activities (trainings, youth survey, strategic financing, priority identification, program selection, capacity building, implementation, fidelity monitoring, scale planning) to short-term outputs (increased capacity, policy changes, greater confidence, designated funding), intermediate outputs (shared accountability, public systems investment, greater program availability with fidelity), and ultimate outcomes (improved youth well-being in programs at Year 1+; reduced risk, enhanced protection, improved system indicators at Years 3+; improved developmental outcomes and reduced disparities at Years 5+). Conscientious, persistent attention to increasing equity and inclusion and reducing racial disparities in well-being undergirds all phases of the framework. The horizontal arrows above the columns indicate the hypothesized process over time, showing how initial inputs from the Casey Foundation and each site support sequential activities that, in turn, lead to short-term outputs, intermediate system changes, and ultimately longer-term improvements in youth outcomes, risk, and protective factors at the community level. Horizontal arrows at the bottom emphasize that conscientious, persistent attention to equity, inclusion, and reducing racial disparities is intended to span and inform all phases of the framework. Adapted from Chilenski et al. [3].

**Table 1.** Alignment of Asset-Based Community Development Principles with Evidence2Success Components.

ABCD Principle	Evidence2Success Component	Illustrative Manifestation in Practice

Citizen leadership and inclusive governance	Resident–agency Community Board	Residents hold voting power in setting priorities and selecting programs
Systematic asset mapping	Youth Experience Survey + community asset scans	Data walks surface informal mentoring groups, faith-based tutoring, and local associations as implementation partners
Social-capital weaving	Cross-sector Board and neighborhood teams	New resident–agency ties secure donated after-school space; horizontal networks connect parent groups
Resource re-orientation	Pooled financing strategy	Parks department budget redirected to resident-prioritized sports league; braided funding from multiple agencies
Inside-out development	Technical assistance engages with the board after local goals are set	External funders and consultants are invited only after residents’ priorities are endorsed by the Community Board
Continuous learning	Quarterly feedback memos and wave interviews	Boards review fidelity data and adjust program supports while maintaining evidence-based core components

*Note.* This table maps the six core principles of Asset-Based Community Development (ABCD) identified by Kretzmann and McKnight [6] and elaborated by Green and Haines [11] onto the corresponding structural components and practices within the Evidence2Success framework. The “Illustrative Manifestation” column provides concrete examples drawn from case study data across Kearns, Mobile, and Memphis communities.

**Table 2.** RQ1—Asset-mapping and local priorities: Illustrative quotations by theme and community.

Theme	Community	Illustrative Quotation

**Existing place-based coalitions and early vision**	Memphis	“The Women’s Foundation for Greater Memphis was fortunate that there was already a structure of people coming together in the South City community… It was our Vision 2020 strategic plan… we were going to invest all of our grant making into one zip code that had high rates of poverty… we pulled together… not-for-profit organizations, faith community representatives, local government, the health department.”
	Mobile	“When I think about how we pitch the community board… we basically said… this is an opportunity for us to take the work that we were already doing around youth outcomes… to the next level… to select things that are evidence-based and not just do things we think are good… and position us for additional resources… to collaborate on grants and partnerships.”
	Kearns	“There was kind of a grassroots effort… around 2008, 2009… community members getting together and saying, okay, we need to figure out something better… the local police department wanted to police us out of some of the challenges… the schools… relegated those kids to more of a remedial track… the kids didn’t get the help they needed.”

**Using data and assessment to sharpen priorities**	Memphis	“There was a needs assessment done with the subset of residents that resided in concentrated public housing… that gave us a really good glimpse into what the needs of that community… they told us things that they needed for their children and some of their challenges with mental health… that really did inform our strategies.”
	Mobile	“After the data came back from the YES survey and folks began to see what young people were saying about their shared experiences, it got real again… we’ve got to put things in place to help respond to it… the initial pitch and the reality began to sync up… folks began to have a shared vision for action.”

*Note.* Interview quotations are drawn from interviews with community board leadership. All quotations are de-identified and presented as illustrative examples of themes identified through cross-case qualitative analysis, rather than as exhaustive representations of each interview.

**Table 3.** RQ2—Governance, social capital, and inclusive processes: Illustrative quotations by theme and community.

Theme	Community	Illustrative Quotation

**Backbone and collective-impact governance**	Memphis	“We were really already implementing a collective impact model that had that shared vision and… the interdependence of the different players and their roles in accomplishing the shared goal.”
	Mobile	“When I think about how we pitch the community board… this is an opportunity for us to take the work that we were already doing around youth outcomes… to the next level… and select things that are evidence-based… and position us for additional resources… to collaborate on grants and partnerships.”
	Kearns	“We had [name removed] as our initial chair… he was told because he was a county employee… it was a conflict of interest… so I took over as chair… we wanted to make sure that people understood that any ideas that came forward… we were going to respect where it was coming from… we created an environment that was safe… with ground rules on how we’re going to conduct ourselves as a coalition.”

**Inclusive processes, voice, and power**	Memphis	“Being intentional about the community board… including those from underrepresented communities and giving them a true equitable voice… not just a meeting where you… listen the whole time, but allowing them to bring whatever gifts and value they had and really… using their input.”
	Mobile	“The first level was… people understanding that we’re all in this together and that this [E2S] is going to give us tools… But that didn’t mean that people felt like they had equal power… it took a lot longer for folks to trust that they could push back on systems or that their voice would carry equal weight.”
	Kearns	“The boards that I’ve served on in the past had a prescribed approach… making sure that people understood that we’re going to respect opinions… we’re not going to disrespect anybody… we wanted the folks to be able to come forward with what they felt… voices got heard… I didn’t turn anybody off, even though sometimes it may have been a little painful to hear some of the folks sharing.”

*Note.* Interview quotations are drawn from interviews with community board leadership. All quotations are de-identified and presented as illustrative examples of themes identified through cross-case qualitative analysis, rather than as exhaustive representations of each interview.

**Table 4.** Leveraged Funds in Memphis Through Strategic Financing and Fundmapping (2015–2020).

Funding Source	Amount (USD)	Purpose

Memphis Shelby County Schools	$262,517	Supporting implementation of Cognitive Behavioral Intervention for Trauma in Schools (CBITS)
Kresge Foundation	$300,000	Evidence2Success infrastructure and coordinator support
Women’s Foundation for a Greater Memphis	$141,067	Strong African American Families (SAAF) implementation ($68,567); Evidence2Success infrastructure ($72,500)
Anonymous donor	$75,000	Evidence2Success infrastructure
Total leveraged	$778,584	

*Note.* This table documents funds secured by the Memphis South City Community Board through strategic financing activities during Years 2–5 of Evidence2Success implementation. “Leveraged” indicates funds redirected from existing allocations or secured from new sources based on Community Board priorities identified through the Youth Experience Survey and local asset-mapping activities. CBITS and SAAF are evidence-based programs selected by the Board to address trauma exposure and family strengthening, respectively. Infrastructure costs include coalition coordinator salary, meeting space, data collection, and technical assistance.

**Table 5.** RQ3—Financing and reallocation of resources: Illustrative quotations by theme and community.

Theme	Community	Illustrative Quotation

**Using Evidence2Success tools to see and realign existing funds**	Memphis	“We use… outcome data from surveys and things that Seeding Success had provided… to put into proposals to our local City of Memphis Housing and Community Development… they did award a grant for us to deliver Safe programming… that’s how we were able to support some of those eight cohorts.”
	Mobile	“The fundmapping process actually helped us see where we were already spending money that could be aligned with what young people said they needed, instead of always looking for a brand-new grant… it meant asking, ‘Are we willing to move money from things we’ve always done to things the community is saying we should do?’”
	Kearns	“We had to sit down with our partners and say, okay, what are we already funding in Kearns and how does that line up with what the YES survey and the community are telling us?… we realized there were some things that we were funding that weren’t necessarily tied to those priorities, and other things we wanted to do but didn’t have a line item for yet.”

**Limits and burdens of braided financing and sustainability**	Memphis	“Even when braiding funding from CDBG… or County funds—some of those sources are not as sustainable… a labor-intensive way to fund the program… one thing I learned… certain types of funding may not work as well as intended.”
	Mobile	“You can braid money to get something started, but if the underlying budgets don’t change, you’re always going to be… trying to hold together a patchwork. That’s exhausting for staff and for community members who are trying to lead from the front.”
	Kearns	“Some of the funding that we’ve relied on has been year-to-year… we can’t always guarantee that the programs we start will still be there three years from now… It’s hard to ask families to trust in something that feels temporary… and hard to ask board members to champion something when they don’t know if the money is going to be there.”

*Note.* Interview quotations are drawn from interviews with community board leadership. All quotations are de-identified and presented as illustrative examples of themes identified through cross-case qualitative analysis, rather than as exhaustive representations of each interview.

**Table 6.** RQ4—Resident knowledge, adaptation, and fidelity: Illustrative quotations by theme and community.

Theme	Community	Illustrative Quotation

**Feedback loops and adaptation decisions**	Memphis	“The people who are implementing your program need to be at the table letting you know what’s going well, what’s not… us having a teachable and a growth mindset, like, okay, is there room to pivot? Is there room to grow?… that’s how we’ve been able to do it and how we will continue to do things.”
	Memphis	“At the Safe reunion, we listened to our community members… facilitators… implementers, and we were like, okay, we need to collect more information… the retention portion of this program.”
	Mobile	“In our organizing work, we’re constantly in conversation with young people and parents about what’s actually happening in schools and neighborhoods… that feedback doesn’t just sit in a notebook; it shapes what we bring back to the board and to system leaders when we’re talking about what needs to change.”

**Limits and gaps in resident engagement**	Memphis	“We’re still trying to figure out how to keep youth at the center of the conversation, not just as a data point but as people who are shaping what we do… We’ve had moments of that, but I wouldn’t say we’ve cracked the code yet.”
	Kearns	“The lack of community engagement… we are not getting the traction with the community because we just have never executed the plan… Right now, we don’t have [a board that] looks like everybody in the community… we have not done what we said we’re going to do as far as sharing who we are as a coalition, what we’re doing and why, how they could benefit, and how they can contribute.”

*Note.* Interview quotations are drawn from interviews with community board leadership. All quotations are de-identified and presented as illustrative examples of themes identified through cross-case qualitative analysis, rather than as exhaustive representations of each interview.

## Data Availability

The data that support the findings of this study are available on request from the corresponding author. The data are not publicly available due to privacy or ethical restrictions. If this manuscript is accepted, we will work with our institution, our funder, and the communities involved in data collection to make the data as available as we can with appropriate protections.

## Footnotes

1 “Resident” refers to community members who live in the focus neighborhoods and hold formal or informal leadership roles (e.g., parents, youth, faith leaders, neighborhood association members), as distinct from staff of public agencies or external organizations.

2 These case studies focus on three Evidence2Success communities (Kearns, Mobile, and Memphis) that had at least two years of framework implementation and sufficient evaluation data; the findings may not represent the full range of experiences in other Evidence2Success sites or in communities using different prevention frameworks.

3 Asset-mapping in these sites combined structured activities (e.g., facilitated walks, mapping workshops, data walks) with informal conversations; examples in the text are illustrative rather than exhaustive of all activities undertaken in each community.

4 Quotations from community members and partners are drawn from evaluation interviews, Strengths and Concerns reports, and Action Guides; minor edits were made to improve readability while preserving the substantive meaning of participants’ statements.

5 Descriptions of coalition structures and decision-making processes reflect the period covered by the process evaluation; some communities may have further adapted their governance arrangements after the timeframe of this study.

6 The term “fundmapping” is used to describe local analyses of existing public and private funding streams that could be realigned to support community-prioritized outcomes; these exercises were conducted collaboratively by community boards, coordinators, and technical assistance providers.

## Abbreviations

ABCD: Asset-Based Community Development
CBITS: Cognitive Behavioral Intervention for Trauma in Schools
CTC: Communities That Care
LDIPN: Lower Dauphin Island Parkway Neighborhood
MLKN: Martin Luther King Neighborhood
MSA: Metropolitan Statistical Area
REEI: Racial and Ethnic Equity and Inclusion
SAAF: Strong African American Families
TA: Technical Assistance
YES: Youth Experience Survey
